# The Problem of Questionable Dystonia in the Diagnosis of ‘Essential Tremor-Plus’

**DOI:** 10.5334/tohm.539

**Published:** 2020-08-13

**Authors:** Sanjay Pandey, Sonali Bhattad, Mark Hallett

**Affiliations:** 1 Department of Neurology, Govind Ballabh Pant Postgraduate institute of medical education and research, New Delhi, IN; 2 Human Motor Control Section, Medical Neurology Branch, National Institute of Neurological Disorders and Stroke, National Institutes of Health, Bethesda, MD, US

**Keywords:** Essential tremor, Dystonic tremor, Dystonia, Neurophysiology

## Abstract

In a recent consensus statement on tremor, the task force of the International Parkinson and Movement Disorder Society proposed a new term, ‘essential tremor-plus (ET-plus)’ which includes patients with the characteristics of essential tremor (ET) and additional soft neurological signs of uncertain significance such as questionable dystonic posturing. The clinical interpretation of questionable dystonia has been left to the investigator. The consensus statement also stated that the ET-plus syndrome does not include other clearly defined syndromes like dystonic tremor. However, the boundary between questionable dystonia and definite dystonia is not distinct leading to diagnostic uncertainty in a clinical setting. A similar case may be classified as ET-plus by one observer and dystonic tremor by another. Following the new definition, many studies have reclassified their ET cohort, and they have highlighted the problem of defining questionable dystonia in the diagnosis of ET plus. ET-plus is likely to be a mixture of patients that actually have dystonia and those that don’t, and clinically all we can do is to be suspicious that there might be dystonia. For example, it is not clear whether we should consider spooning and index finger pointing as a sign of questionable or definite dystonia. There are major research and possible therapeutic implications of questionable dystonia in the diagnosis of ET-plus. The concept of ET-plus is extremely difficult to implement without definite guidelines. The resolution will need a biomarker such as physiology or imaging.

## Introduction

Essential tremor-plus (ET-plus) is a new term proposed in the recent classification of tremor [1]. Tremor with the characteristics of essential tremor (ET) and additional neurological signs of uncertain significance such as questionable dystonic posturing has been classified as ET-plus [1]. If a patient had definite dystonia that should be classified as dystonic tremor or tremor associated with dystonia, but the boundary between questionable dystonia and definite dystonia is not distinct. This new terminology was introduced to improve the phenotyping of patients so that more homogeneous subgroups would be created that might lead to advances in understanding etiologies and in developing therapies.

## ET-plus likely is a mixture of patients that actually have dystonia and those that don’t

Dystonia in ET cohorts have been frequently (range; 0–47%) reported and the most common reported dystonias were blepharospasm, neck dystonia, and focal hand dystonia [2,3]. According to the previous consensus statement of the Movement Disorder Society on Tremor (1998) one of the major exclusion criteria for the diagnosis of ET was the presence of dystonia [4]. However, dystonia was still being reported in the majority of ET cohorts [5,6,7,8]. The term ‘questionable dystonia’ was a new addition in the new tremor classification in 2018 and subsequently, many researchers have published retrospective analyses of their existing ET cohorts (Table 1) [9,10,11,12,13]. A significant number of patients were reclassified as ET-plus due to the presence of questionable or mild dystonia. We can make some important conclusions from these studies. First, after applying the new diagnostic criteria, pure ET is very likely less common than ET-plus. Second, applying the ET-plus criteria including “questionable dystonic posturing” is challenging with a high rate of discordance. Third, ET-plus patients who were thought to have questionable dystonia were significantly older and had a longer duration of disease than ET patients. Fourth, greater upper limb action tremor and tremor spread to the cranial region were significantly associated with ET-plus.

**Table 1:** Studies reclassifying essential tremor patients due to questionable dystonia/dystonia.

Author/year	Number of ET patients re-evaluated	*Total number of patients re-classified as ET-plus using 2018 consensus criteria	Number of patients with ET-plus because of questionable dystonia/dystonia	Comments

Rajalingam 2018 [9]	133	110	5/0	The results of the study may not apply well to a more typical group as patients were selected on the basis of having lower limb tremor
^10^Prasad 2019 [10]	252	99	21/0	Dystonia was referred to as mild
^11^Pandey 2019 [11]	79	31	19/0	Dystonia was labelled as questionable if there was discordance between the examiners regarding its presence
^12^Huang 2019 [12]	280	117	0/0	Neither certain nor questionable dystonia was seen in any patient
Amlang 2020 [13]	104	0	0/29	Investigators used the 1998 consensus criteria so did not make a diagnosis of ET-plus, but made the diagnosis of DT

* Note that the first four studies in the table did not identify any patient with definite dystonia, and the fourth study did not even identify any patient with questionable dystonia. The fifth study identified 29 patients with definite dystonia, but did not consider the possibility of questionable dystonia.

## Clinically all we can do is to be suspicious that there might be dystonia

The findings from these recent studies are consistent with the previous reports that soft signs including dystonia are challenging in the diagnosis of ET-plus. However, there are no quantification tools to measure the “questionable dystonia” and subtle clinical features of dystonia described in forms of “spooning” and “Index finger-pointing (IFP)” further add to the diagnostic uncertainty. “Spooning” was defined as wrist flexion and metacarpophalangeal hyperextension whereas “IFP” was defined as an extension of the index finger and partial or full flexion of the other digits [14,15]. Kim and colleagues proposed that recognition of “spooning” during the evaluation of tremor may aid in the diagnosis of underlying dystonia, but overextending the arms may sometimes produce a posture that resembles spooning [14]. Vives-Rodriguez and Louis have recently reported that IFP during walking may be a subtle form of dystonia [15]. They enrolled 250 patients including PD (n = 50), dystonia (n = 50), ET (n = 80) and healthy controls (n = 70) and reported IFP in 11.6% (29/250) participants. The highest prevalence (20%) of IFP was seen in patients with idiopathic dystonia, followed by PD (16.0%), ET (10%) and healthy controls (3.8%) [15]. Although the differences were small, the authors concluded that subtle dystonic posturing such as IFP may be an important diagnostic clue towards underlying dystonia [15].

## We do know the research implications of questionable dystonia, but not the therapeutic implications

**Figure 1:**
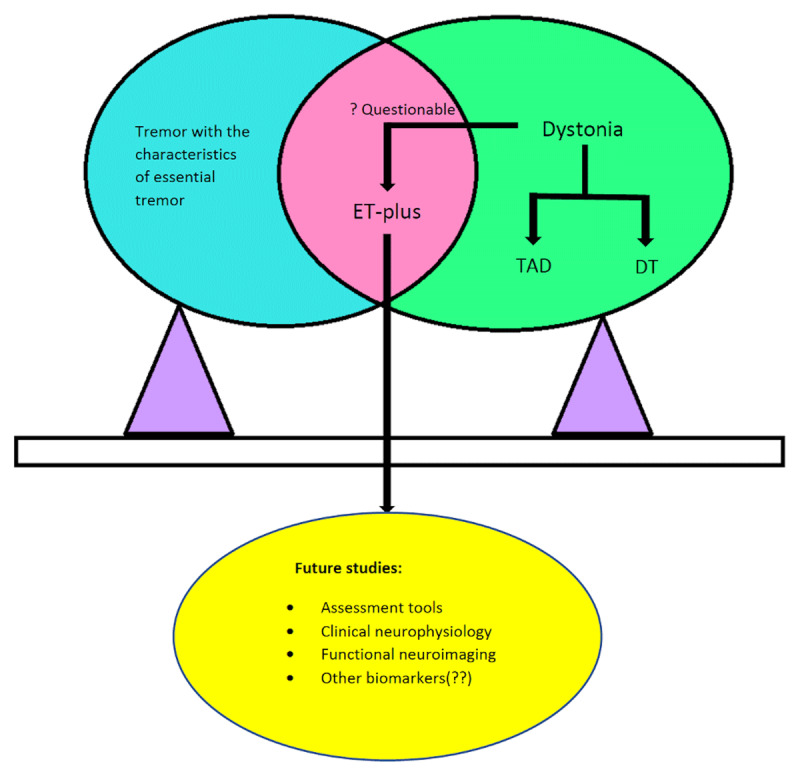
Tremor with characteristics of Essential tremor along with the presence of questionable dystonia is classified as ‘Essential tremor-plus (ET-plus)’, whereas tremor with definite dystonia can be further classified into tremor associated with dystonia (TAD) and dystonic tremor (DT). Future studies are needed to define the “questionable dystonia” in ET-plus which can be obtained through various assessment tools, clinical neurophysiology, and various biomarkers which are to be yet identified.

In the absence of a clear definition of questionable dystonia, when an investigator is doing a study of ET-plus, some of the patients would have been diagnosed as ET or dystonic tremor by other investigators (Figure 1) [16]. In longitudinal cohort studies, some ET-plus patients at the baseline may be diagnosed as DT during the follow-up assessment, and it will be interesting to know what percentage of patients have that course. Genetic studies in ET-plus patients are challenging considering the phenotypic heterogeneity present in the same family and the dystonia may be classified as questionable in one family member and definite in another family member. It is very important when doing family studies looking for mutations to avoid false-positive cases.

Defining questionable dystonia in ET-plus patients has possible therapeutic implications also. Following medical treatment tremor symptoms are reduced in 50% of ET patients only, rarely complete disappearance is observed [17,18]. It may be possible that many of the earlier studies may have recruited some dystonic tremor patients also in their ET cohort, leading to unsatisfactory outcomes in around half of the patients.

## Is there a common link between the pathogenesis of ET-plus and dystonic tremor? Maybe it is the cerebellum

The pathogenesis of DT remains speculative, but the available neurophysiological findings indicate that tremor has no influence on the basic pathophysiological features of dystonia [2]. Traditionally dystonia has been considered as a basal ganglia disorder, but it is now regarded as a ‘network disorder’ including the cerebellum [19,20,21]. Studies have shown both clinical observations that a cerebellar lesion can evoke dystonic features and behavioral, neurophysiological, and functional imaging results that establish a firm link between the cerebellum and dystonia [22,23,24]. In one study, cervical dystonia patients with tremor showed abnormal anticipatory performance in a multi-joint catching task when compared to cervical dystonia patients without tremor or control subjects, suggesting a possible cerebellar influence on the expression of clinical phenotypes in dystonia [25]. The role of the cerebellum in the pathogenesis of ET has been postulated based on clinical, neuroimaging, and pathological studies [23]. So, we can hypothesize that the common features of ET and DT pathogenesis may arise in the cerebellum.

## ET-plus or dystonic tremor: The resolution will need a biomarker such as physiology or imaging

The previous (1998) consensus statement proposed to define dystonic tremor syndromes under two groups, dystonic tremor (DT: tremor in dystonic body segment) and tremor associated with dystonia (TAD: tremor in non-dystonic body segment) [4]. The new classification (2018) retained these but stated that there is no reason to suspect a different etiology for the TAD, even though such tremor might be regarded as a form of ET if the dystonia was not present [1]. To address the situation faced by clinicians in classifying a patient who has ET for decades before developing dystonia, they noted that the classification of such patients should be “dystonic tremor with antecedent ET”. Clinical syndromes can evolve. Fasano and colleagues later criticized the classification by stating that this is the dual disease pitfall, very similar to the controversial “ET-PD” issue [26].

Certain clinical features such as irregular head tremor with directional quality persisting during supine position and presence of sensory trick and null point indicate a dystonic tremor and may help in differentiating these patients from ET [2,27]. Also, on handwritten spirals a single predominant axis is more often observed in ET than DT [28]. The consensus classification notes that electrophysiological tests may help in resolving the ET-plus and DT controversy.

Surface electromyography with an accelerometer has been used where tremor irregularity is frequently observed in DT patients compared with ET patients [29]. Blink reflex recovery curve is a measure of brainstem excitability and studies have shown increased R2 in DT patients compared to ET patients [30]. Patients with dystonic tremor have increased somatosensory temporal discrimination threshold (STDT) which is defined as the shortest time interval in which subjects can perceive two stimuli as being separated [31]. In comparison, STDT is normal in ET patients and healthy controls. However, in a clinical setting, using electrophysiological tests may be difficult to do as they are not available easily, they may not be helpful in an individual patient, and they are not validated for understanding ET-plus. It will be important to take patients with ET-plus and test them to see how often the tests are normal or abnormal. Different neuroimaging studies have also been used as a possible biomarker for ET and DT patients, but the results are non-specific [32,33,34,35].

## There are other problems with ET-plus apart from questionable dystonia

Other neurological signs of uncertain significance such as impaired tandem gait and memory impairment have also been included in the definition of ET-plus [1]. It is true that patients with ET accumulate these additional clinical features during the course of disease [16]. Also, patients with ET might later develop rest tremor. So, ET-plus might only represent a state condition rather than a trait condition. Patients with ET might evolve to ET-plus. We do not know at this time whether these additional neurological signs are disease-linked or coincidental age-related or symptomatic of additional Parkinson’s disease pathology [36].

## The concept of ET-plus is difficult to implement without guidelines

How much of a neck tilt is dystonia vs. normal variation? Can it be defined in degrees? It might actually require some sophisticated clinical neurophysiology to separate the types. We need to develop an assessment tool, video protocol, and scales to capture subtle or questionable features of dystonia which will guide the clinician in deep phenotyping to distinguish ET-plus from ET and dystonic tremor. Certain tremor characteristics (e.g., irregularity in amplitude and rhythm, posturing of the head or limb, fluctuating axis of rotation) are believed to be useful in differentiating ET-plus and dystonic tremor patients [27,37]. Computerized video analysis, motion transducers, and electrophysiologic techniques may also be helpful in the assessment of clinically challenging patients.

The real challenge in accepting the new terminology is to label the dystonia as questionable, mild, or definite. In the absence of a clear definition, the boundaries between dystonic tremor, tremor associated with dystonia and ET-plus remain blurred. Further research is required in electrophysiology, neurostimulation, functional brain imaging, focus ultrasonography, and biological markers to get more insights into this new terminology. Of course, it would have been good if the original committee could have agreed on where to draw the line, but they could not. The problem with questionable signs is that they are questionable.

This needs resolution. To make the diagnosis more valuable the questionable signs need to be categorized and quantified. How much head tilt in angle and percent time should be considered definite dystonia, questionable, and within normal limits? Should spooning and index finger pointing formally be included as ET+? This is a difficult but not impossible task.
